# *CMJ* – Continuity through changes

**DOI:** 10.3325/cmj.2019.60.67

**Published:** 2019-04

**Authors:** Svjetlana Kalanj Bognar

**Affiliations:** 1 *Croatian Medical Journal*, Zagreb, Croatia; 2 University of Zagreb School of Medicine, Zagreb, Croatia *svjetlana.kalanj.bognar@cmj.hr*

Working as an editor of a medical journal is quite a challenging duty. It requires personal commitment and motivation, experience in writing and reviewing scientific articles, publishing know-how, and probably a great deal of diplomacy and common sense in decision-making. Newly appointed editors-in-chief are confronted with an immense responsibility toward authors and reviewers, the entire critical professional audience, but also toward their editorial teams (1). Maintaining and increasing the journal’s quality and introducing new ideas in order to expand its visibility and recognition within the international scientific community are not easy tasks. In addition, there is the huge obligation to maintain the accomplishments of previous editors-in-chief.

Knowing all that, in the spring of 2019, I took over the honorable duty of the editor-in-chief of the *Croatian Medical Journal* (*CMJ*). I remember the *CMJ*’s beginnings and have been witnessing its progress throughout my whole career as a scientist and teacher at the Zagreb School of Medicine – the *CMJ* was founded in 1992, the year in which I was employed as a PhD fellow at the Department for Chemistry and Biochemistry. In fact, as a naive and idealistic young scientist, I submitted a manuscript for publication in the *CMJ*. Although this attempt was not successful, I received unexpectedly constructive critical comments and detailed editorial suggestions for manuscript improvement. Not only did I publish a substantially improved article in a journal with a higher impact factor, but above all, this experience made me realize the immense importance of journal’s educational role and its editor's obligation to strive for maximum manuscript quality and originality.

The beginnings of the *CMJ* were hard enough, but a journal that started as a publication venue for scientists from developing countries has evolved significantly and admirably. The *CMJ* is today a modern journal covering broad aspects of biomedicine, with transparent editorial policy and an important advantage of providing open access to authors and readers. Moreover, it is a journal in continuous pursuit of excellence in every issue (2). Realistically – yes, we are a small journal, and yes, we cannot compare our production with giants in medical publishing business. However, our constraints may turn out to be our strength. I believe that by a smart editorial policy we may attract original and well-written manuscripts within specific and recognizable research niches. Are we supposed to frantically run for higher and higher bibliometric score? Or, should we concentrate on what is really important in science – to ask new questions, to recognize new scientific ideas, to be open to new paradigms, and at the same time be critical and aware of our responsibility to society? I believe that in the long-term such a policy may prove to be beneficial for our journal, which is now in its mature years and ready to achieve its full potential (3). The important premises are here – a team of enthusiastic editors with more or less experience in publishing, respectable members of Managerial, Editorial, and Advisory boards, and administrative support of three Croatian Medical Schools. As presented previously by Anton Glasnović, my associate and deputy-in-chief, we will proceed with drawing the attention of potential authors by optimizing manuscript managing procedure and we will continue nurturing the journal’s reputation and quality (4). In 2019, the *CMJ* publishes two thematic issues, and one of them is just in front of you. Being a neuroscientist, I am particularly happy to write my first editorial in the issue dedicated to the “International Conference on Neurological Disorders and Neurorestoration,” which takes place in May 2019 in the inspiring renaissance scenery of Dubrovnik. By becoming the *CMJ*’s editor-in-chief, I got the opportunity to observe the other side of manuscript managing and publication. At the same time, I took over a serious but fulfilling task that I intend to perform with all my heart and with full assistance of my editorial team.
